# Chamomile decoction extract inhibits human neutrophils ROS production and attenuates alcohol-induced haematological parameters changes and erythrocytes oxidative stress in rat

**DOI:** 10.1186/s12944-016-0233-4

**Published:** 2016-03-31

**Authors:** Mohamed-Amine Jabri, Mamane Sani, Kais Rtibi, Lamjed Marzouki, Jamel El-Benna, Mohsen Sakly, Hichem Sebai

**Affiliations:** Laboratoire de Physiologie Intégrée, Faculté des Sciences de Bizerte, 7021 Zarzouna, Tunisia; Laboratoire de Physiologie Fonctionnelle et Valorisation des Bio-Ressources - Institut, Supérieur de Biotechnologie de Béja, Université de Jendouba, Avenue Habib Bourguiba, B.P. 382-9000 Béja, Tunisia; UMR Biosurveillance et Toxicologie Environnementale, Département de Biologie, Faculté des Sciences et Techniques de Maradi, 465 Maradi, Niger; INSERM, U1149, Centre de Recherche Sur l’Inflammation - Faculté de Médecine X. Bichat, 75018 Paris, France

**Keywords:** Chamomile, Ethanol, Haematological parameters, Oxidative stress, Rat

## Abstract

**Background:**

The aim of this study was to evaluate the protective effects of subacute pre-treatment with chamomile (*Matricaria recutita* L.) decoction extract (CDE) against stimulated neutrophils ROS production as well as ethanol (EtOH)-induced haematological changes and erythrocytes oxidative stress in rat.

**Methods:**

Neutrophils were isolated and ROS generation was measured by luminol-amplified chemiluminescence. Superoxide anion generation was detected by the cytochrome c reduction assay. Adult male wistar rats were used and divided into six groups of ten each: control, EtOH, EtOH + various doses of CDE (25, 50, and 100 mg/kg, *b.w.*), and EtOH+ ascorbic acid (AA). Animals were pre-treated with CDE extract during 10 days.

**Results:**

We found that CDE inhibited (*P* ≤ 0.0003) luminol-amplified chemiluminescence of resting neutrophils and N-formyl methionylleucyl-phenylalanine (fMLF) or phorbolmyristate acetate (PMA) stimulated neutrophils, in a dose-dependent manner. CDE had no effect on superoxide anion, but it inhibited (*P* ≤ 0.0004) H_2_O_2_ production in cell free system. In vivo, CDE counteracted (*P* ≤ 0.0034) the effect of single EtOH administration which induced (*P* < 0.0001) an increase of white blood cells (WBC) and platelets (PLT) counts. Our results also demonstrated that alcohol administration significantly (*P* < 0.0001) induced erythrocytes lipoperoxidation increase and depletion of sulfhydryl groups (−SH) content as well as antioxidant enzyme activities as superoxide dismutase (SOD), catalase (CAT), and glutathione peroxidase (GPx). More importantly, we found that acute alcohol administration increased (*P* < 0.0001) erythrocytes and plasma hydrogen peroxide (H_2_O_2_), free iron, and calcium levels while the CDE pre-treatment reversed increased (*P* ≤ 0.0051) all these intracellular disturbances.

**Conclusions:**

These findings suggest that CDE inhibits neutrophil ROS production and protects against EtOH-induced haematologiacal parameters changes and erythrocytes oxidative stress. The haematoprotection offered by chamomile might involve in part its antioxidant properties as well as its opposite effect on some intracellular mediators such as H_2_O_2_, free iron, and calcium.

## Background

Reactive oxygen species (ROS) are involved in a wide range of processes, such as aging and diseases [1]. Nevertheless, ROS are not only an aspect of normal metabolism but are also implicated in several physiological phenomena such as the substantial protection against severe infections [2] and the redox regulation of protein phosphorylation, ion channels, and transcription factors [3]. However, the enhanced ROS production may lead to oxidative stress and oxidation of vital cellular components, which induce cellular damage and cell death [4]. Therefore, the cytotoxicity of ethanol was attributed to increased ROS generation [5, 6], which in turn consequently leaded to injuries and oxidative stress in many organ systems [7]. To protect cells against these harmful species we use a synthetic or natural antioxidants molecules [8, 9]. These latter are able to scavenge ROS and to up-regulate endogenous antioxidant defense systems [10]. Mammalian erythrocytes are endowed with extraordinarily efficient enzymatic and non-enzymatic antioxidant defense systems that act as ROS scavengers to limit their imposed damage [11]. The importance of the protective mechanisms of erythrocytes is evident from a consideration of human haemolytic disorders due to a variety of enzyme deficiencies involving pathways that maintain intracellular reductive molecules [12]. Deficiencies compromising the capacity to detoxify oxidant molecules such H_2_O_2_ and O_2_^•-^ radicals result in oxidant-induced denaturation of intracellular molecules and premature destruction of erythrocytes. Nevertheless, despite the limited biosynthetic repertoire available to mature erythrocytes, they are resilient to oxidant-induced damage. Clearly, antioxidants in the form of scavengers and detoxifying enzymes provide an important protective system in erythrocytes [12]. However, erythrocytes are considered as passive ‘reporter cells’ for the oxidative status of the whole organism and an increasing amount of attention is being paid to the use of plant molecules such as polyphenolic and carotenoid components [13] in the prevention and cure of various [13].

Chamomile (*Matricaria recutita* L.) is a medicinal plant belonging to Compositae family. It is one of the ancient and most popularly consumed beverages worldwide, including Tunisia [14]. This species is traditionally known for its beneficial effects for the treatment of hepatic and gastrointestinal disorders such as diarrhea [15–17]. From the experimental and clinical studies performed on *Matricaria recutita*, it seems that the majority of its pharmacological actions are related to its antioxidant activity which is mainly due to its ability to scavenge free radicals and/or inhibit lipid peroxidation [17, 18]. For this reason, chamomile extracts are known to exhibit many beneficial health effects as neuro-protective [19], anti-allergic [20], anti-microbial [21], anti-cancer [22], and anti-inflammatory [23].

The present study was undertaken to investigate the protective effect of chamomile decoction extract on haematological parameter disorders and erythrocytes-induced oxidative stress after the acute alcohol administration. We also studied the implication of some intracellular mediators as H_2_O_2_, free Fe, and Ca in such protection.

## Methods

### Chemicals

PMA, fMLF, protease inhibitors, and cytochrome *c* were from Sigma–Aldrich (St Quentin Fallavier, France). Epinephrine, bovine catalase, 2-Thio-barbituric acid (TBA), and butylated hydroxytoluene (BHT) were from Sigma Chemicals Co (Germany). All other chemicals used were of analytical reagent grade.

### Preparation of chamomile decoction extract

Chamomile flowers were collected from the region of Beja (North-West of Tunisia) during March 2013. The plant material was later dried in an incubator at 40 °C during 72 h and powdered in an electric blender. The decoction was made with double distilled water (1/5; w/v) at 100 °C during five minutes under magnetic agitation and the homogenate was filtered through a colander (0.5 mm mesh size). Finally, the obtained extract (CDE) was stored at −80 °C until used.

### Isolation and preparation of human neutrophils

Venous blood was collected from healthy adult volunteers and neutrophils were isolated by Dextran sedimentation and density gradient centrifugation as previously described by El-Benna and Dang [24]. Erythrocytes were removed by hypotonic lysis. Following isolation, the cells were resuspended in Hank’s balanced salt solution (HBSS). The cells were counted and their viability was determined with the trypan blue exclusion method.

### Ethics

Neutrophils were isolated from venous blood of healthy volunteers managed in the hematology and immunology department of Bichat Hospital, Paris, France. The investigations were approved by the local ethics committee and samples were obtained with the volunteers’ and patients’ written informed consent. All experiments were approved by the ‘Institut National de la Santé et de Recherche Médicale (INSERM)’ institutional review board and ethics committee. Data collection and analyses were performed anonymously.

### Measurement of ROS production by chemiluminescence

Isolated cells were resuspended in HBSS at a concentration of 1 million per mL. Cell suspensions (5 × 10^5^) in 0.5 mL of HBSS containing 10 μM luminol in the presence or absence of CDE were preheated to 37 °C in the thermostatted chamber of a luminometer (Berthold-Biolumat LB937) and allowed to stabilize. After a baseline reading, cells were stimulated with 0.1 μM fMLF or 100 ng/mL PMA. Changes in chemiluminescence were measured over a 30-min period.

### Measurement of superoxide anion production

Isolated cells were also resuspended in HBSS at a concentration of 1 million per mL. Cell suspensions in 1 mL of HBSS containing 1 mg/mL cytochrome *c* in the presence or absence of CDE were preheated to 37 °C in the thermostatted chamber of a spectrophotometer (Uvikon) and allowed to stabilize. After a baseline reading, cells were stimulated with 0.1 μM fMLF or 100 ng/mL PMA. Changes in absorbance were measured at 550 nm over a 15-min period.

### Measurement of H_2_O_2_ inhibition by chemiluminescence

The effect of CDE on H_2_O_2_ was tested in a cell free system using horseradish peroxydase (HRPO). The reaction mixture contained 10 μM luminol in the presence or absence of MBSAE. The reaction was started by addition of 2.5 U/mL horseradish peroxydase (HRPO), and lucigenin chemiluminescence was measured at 37 °C for 30 min in a luminometer (Berthold-Biolumat LB937).

### Animals and treatment

Healthy adult male Wistar rats (200–220 g body weight- 15 weeks old) were purchased from the Pasteur Institute of Tunis and used in accordance with the local ethics committee of Tunis University for the use and care of animals in accordance with the NIH recommendations. They were provided with standard food (standard pellet diet- Badr Utique-TN) and water *ad libitum* and maintained in animal house at controlled temperature (22 ± 2 °C) with a 12 h light–dark cycle. The rats were divided into half a dozen groups of 10 animals each. Groups 1 and 2 served as controls and received bidistilled water. Groups 3, 4, and 5 were pre-treated with various doses of CDE (25, 50, and 100 mg/kg, *b.w. p.o.*) while group 6 received ascorbic acid (250 mg/kg, *b.w. p.o.*). Animals were pre-treated during 10 days. After 60 min of the last administration, each animal, except those of group 1, was intoxicated by acute oral administration of EtOH (4 g/kg, *B.w.*).

### Blood cells count and erythrocytes preparation

Two hours after the EtOH intoxication, 0.5 mL of blood was firstly collected by ocular ponction in EDTA tubes for blood cells count using a haematology analyzer Coulter MAXM (Beckman Coulter, Inc., Fullerton, USA). Then, animals were immediately sacrificed and blood was collected in heparinized tubes. Erythrocytes were isolated by gentle centrifugation (2 000 g , 15 min at 4 °C), resuspended in isotonic phosphate buffer pH 7.4, and lysed with a hypotonic solution consisting of 20 mM Tris–HCl pH 7.2. Obtained homogenates were after used for biochemical determination of protein, free iron, calcium, H_2_O_2_, SH-groups, and MDA levels as well as antioxidant enzyme activities.

### Biochemical estimations

SOD activity was estimated according to the method described by Misra and Fridovich [25]. CAT activity was measured using Aebi’s method [26]. GPx activity was determined according to the method described by Flohé and Günzler [27]. Thiol groups (−SH) was performed according to Ellman’s method [28]. MDA was estimated using the thiobarbituric acid test [29]. H_2_O_2_ was estimated using the method of Dingeon et al.[30]. Erythrocytes non haem iron was measured by colorimetrically using ferrozine as described by Leardi et al.[31]. Calcium was performed according to Stern and Lewis method [32]. The protein content was determined according to Hartree [33] which is a slight change of the Lowry method.

### Statistical analysis

The data were analyzed by one-way analysis of variance (ANOVA) and were expressed as means ± standard error of the mean (S.E.M.). The data are representative of 10 independent experiments. All statistical tests were two-tailed, and a *p* value of 0.05 or less was considered significant.

## Results

### Effect of CDE on luminol-amplified chemiluminescence in human neutrophils

**Fig. 1:**
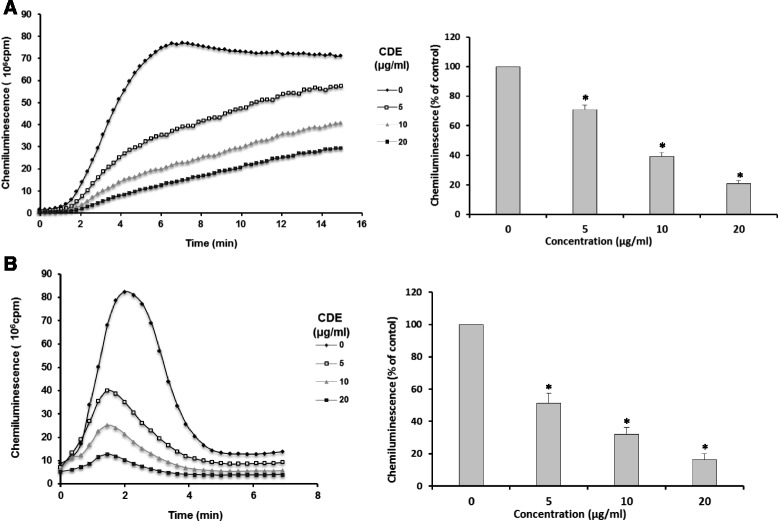
Effect of CDE on luminol-amplified chemiluminescence in human neutrophils. Human neutrophils (5 × 10^5^) were incubated in the presence or absence of different CDE concentrations and stimulated with PMA(**a**) or fMLF (**b**). Luminol-amplified chemiluminescence was measured for 30 min (Data are presented as means ± S.E.M. of five independent experiments, *p < 0.05)

To investigate the antioxidant effect of CDE on human neutrophils, we first looked at the luminol-amplified chemiluminescence stimulated with PMA (Fig. 1a) and fMLF (Fig. 1b) in these cells. Compared with cells not stimulated with any chemical or resuspended in HBSS alone, CDE significantly (*P* ≤ 0.0003) and dose-dependently inhibited luminol-amplified chemiluminescence.

### Effect of CDE on fMLF and PMA-induced neutrophils superoxide anion production

**Fig. 2:**
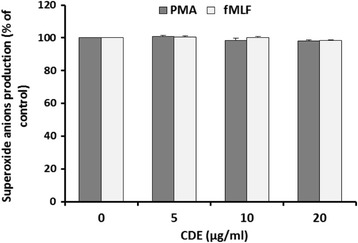
Effect of chamomile decoction extract (CDE) on superoxide anion production by human neutrophils using cytochrome c reduction assay. Cells (1 × 10^6^) were incubated in the presence or not of different CDE concentrations and stimulated with PMA (100 ng/mL) or fMLF (0.1 μM). Cytochrome c reduction was measured at 550 nm in a spectrophotometer for 10 min (Data are presented as means ± S.E.M. of five independent experiments, *p < 0.05)

Next we focused on the experimental production of superoxide anion in human neutrophils treated with fMLF and PMA (Fig. 2). First, we pretreated neutrophils with various concentrations of CDE from 0 to 20 μg/mL. After neutrophils suspension in HBSS solution and their stabilization, they were stimulated with fMLF and PMA. Then, we investigated the change in superoxide anion production induced by fMLF and PMA over time. Although the exposure of neutrophils to various doses of CDE, it does not affect the production of superoxide anion (Fig. 2).

### Effect of CDE on H_2_O_2_ production in a cell free system

**Fig. 3:**
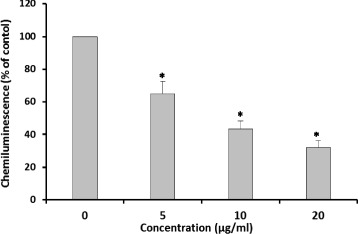
Effect of CDE on luminol-amplified chemiluminescence in the presence or not of different concentrations chamomile decoction extract (CDE) and stimulated with 2.5 U/mL horseradish peroxydase (HRPO). Luminol-amplified chemiluminescence was measured for 30 min (Data are presented as means ± SEM of five independent experiments, *p < 0.05)

To verify the role of CDE on H_2_O_2_ production in a cell free system, we evaluated the luminol-amplified chemiluminescence stimulated with horseradish peroxydase (HRPO) (Fig. 3). CDE dose-dependently showed dramatic and significant (*P* ≤ 0.0004) inhibition of H_2_O_2_ production.

### Effect of EtOH and CDE on haematological parameters

**Fig. 4:**
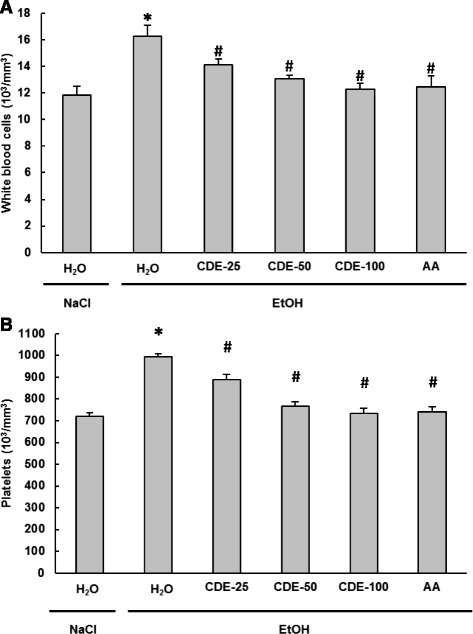
Subacute effect of chamomile decoction extract (CDE) on acute EtOH-induced changes in WBC (**a**) and PLT (**b**) counts. Animals were pre-treated during 10 days with CDE (25, 50 and 100 mg/kg *b.w., p.o.)* or vehicle (bidistilled H_2_O), challenged with a single oral administration of EtOH (4 g/kg *b.w.*) or NaCl 9‰ for 2 h. Assays were carried out in triplicate. *: *p* < 0.05 compared to control group and **#**: *p* < 0.05 compared to EtOH group

The main haematological parameters analysed were WBC and PLT. Both were significantly (*P* < 0.0001) increased after the acute administration (6 g/kg, b.w., p.o.) of EtOH (Fig. 4).

CDE pre-treatment significantly (*P* ≤ 0.0034) and dose-dependently abrogated these haematological deregulations induced by EtOH intoxication, with the same efficiency than ascorbic acid.

### Effect of EtOH and CDE on Erythrocytes lipoperoxidation

**Fig. 5:**
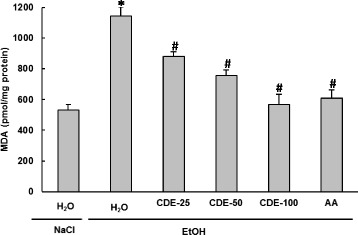
Subacute effect of chamomile decoction extract (CDE) on acute EtOH-induced changes in erythrocytes MDA level. Animals were pre-treated during 10 days with CDE (25, 50 and 100 mg/kg *b.w., p.o.)* or vehicle (bidistilled H_2_O), challenged with a single oral administration of EtOH (4 g/kg *b.w.*) or NaCl 9‰ for 2 h. Assays were carried out in triplicate. *: *p* < 0.05 compared to control group and #: *p* < 0.05 compared to EtOH group

Concerning the effect of EtOH and CDE on oxidative stress condition, we firstly studied the erythrocytes lipoperoxidation (Fig. 5). Acute EtOH administration drastically (*P* < 0.0001) increased the erythrocyte MDA levels. However, CDE and AA pre-treatment significantly (*P* < 0.0001) and dose-dependently reversed lipoperoxidation induced by alcohol intoxication. With the dose of 100 mg/kg the protective effect was similar to that of ascorbic acid.

### Effect of EtOH and CDE on Erythrocytes- SH groups content

**Fig. 6:**
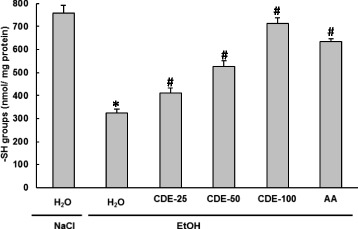
Subacute effect of chamomile decoction extract (CDE) on acute EtOH-induced changes in erythrocytes SH- groups level. Animals were pre-treated during 10 days with CDE (25, 50 and 100 mg/kg *b.w., p.o.)* or vehicle (bidistilled H_2_O), challenged with a single oral administration of EtOH (4 g/kg *b.w.*) or NaCl 9‰ for 2 h. Assays were carried out in triplicate. *: *p* < 0.05 compared to control group and #: *p* < 0.05 compared to EtOH group

In the present study, the effects of EtOH and CDE treatment on SH-group levels were also examined. The data from Fig. 6 showed that acute EtOH administration significantly (*P* < 0.0001) decreased the content of -SH groups. CDE (25, 50, and 100 mg/kg; *b.w.*) pre-handling significantly (*P* ≤ 0.0024) and dose-dependently protected erythrocytes sylfhydryls against depletion caused by alcohol administration. Ascorbic acid pre-treatment, used as reference molecule, also abrogated sulhydryl groups’ decrease.

### Effect of EtOH and CDE on Erythrocytes antioxidant enzyme activities

**Fig. 7:**
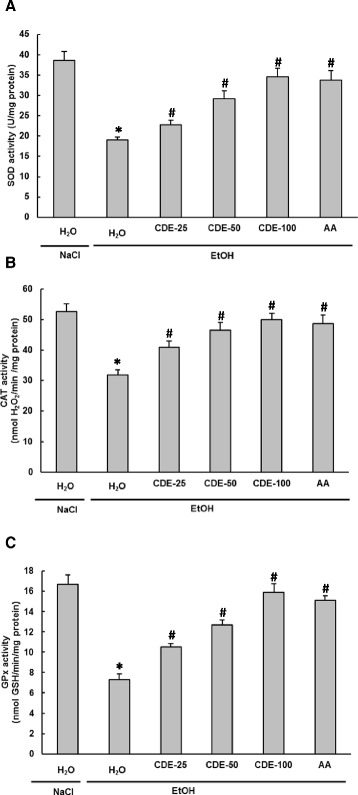
Subacute effect of chamomile decoction extract (CDE) on acute EtOH-induced changes in erythrocytes antioxidant enzyme activities SOD (**a**), CAT (**b**) and GPx (**c**). Animals were pre-treated during 10 days with CDE (25, 50 and 100 mg/kg *b.w., p.o.)* or vehicle (bidistilled H_2_O), challenged with a single oral administration of EtOH (4 g/kg *b.w.*) or NaCl 9‰ for 2 h. Assays were carried out in triplicate. *: *p* < 0.05 compared to control group and #: *p* < 0.05 compared to EtOH group

We further examined the effect of EtOH and CDE on erythrocytes antioxidant enzyme activities (Fig. 7). We showed that alcohol administration significantly (*P* < 0.0001) decreased erythrocytes antioxidant enzyme activities as SOD (A), CAT (B), and GPx (C). CDE pre-treatment significantly (*P* ≤ 0.0051) reversed all EtOH-induced antioxidant enzymes depletion in a dose-dependent manner. Ascorbic acid, an antioxidant reference molecule, also exhibited the same protection.

### Effect of EtOH and CDE on Erythrocytes H_2_O_2_, free iron, and calcium levels

We reported in Table 1 the effect of Ethanol and CDE on intracellular mediators as hydrogen peroxide, free iron, and calcium levels. EtOH *per se* drastically (*P* < 0.0001) increased iron, H_2_O_2,_ and calcium levels. CDE or ascorbic acid pre-treatment significantly (*P* ≤ 0.0051) revealed protective effect against EtOH-induced intracellular mediator disturbances in a dose-dependent manner. CDE at a dose of 100 mg/kg and ascorbic acid at a dose of 250 mg/kg were equally efficient to totally correct these alterations.

**Table 1:** Subacute effect of chamomile decoction extract (CDE) on acute EtOH-induced changes in erythrocytes hydrogen peroxide, free iron, and calcium levels

Groups	H_2_O_2_ (μmol/mg protein)	Free iron (μmol/mg protein)	Calcium (μmol/mg protein)
Control	0.99 ± 0.062	9.04 ± 0.55	220.48 ± 14.25
EtOH	1.85 ± 0.068*	22.72 ± 1.04*	370.15 ± 10.13*
EtOH + CDE-25	1.60 ± 0.054^#^	17.11 ± 1.15^#^	321.22 ± 12.47^#^
EtOH + CDE-50	1.23 ± 0.056^#^	14.37 ± 0.61^#^	293.03 ± 9.38^#^
EtOH + CDE-100	1.09 ± 0.077^#^	11.23 ± 0.54^#^	237.23 ± 17.65^#^
EtOH + AA	1.03 ± 0.041^#^	10.71 ± 0.69^#^	235.18 ± 13.96^#^

Animals were pre-treated during 10 days with CDE (25, 50 and 100 mg/kg *b.w., p.o.)* or vehicle (bidistilled H_2_O), challenged with a single oral administration of EtOH (4 g/kg *b.w.*) or NaCl 9‰ for 2 h. Assays were carried out in triplicate. *: *p* < 0.05 compared to control group and #: *p* < 0.05 compared to EtOH group

## Discussion

The current study was designed to investigate the effect of CDE on human neutrophil reactive oxygen species (ROS) production in vitro as well as to determine its protective effects on EtOH-induced haematological alterations and erythrocytes oxidative stress in rat.

We firstly tested the CDE on human neutrophils total ROS production, in response to chemotactic peptide (fMLF) and phorbolmyristate acetate (PMA) stimulation, as well as on H_2_O_2_ accumulation in a cell free system. Our data showed that CDE (5, 10 and 20 μg/mL) treatment significantly inhibited luminol-amplified chemiluminescence in neutrophils and H_2_O_2_ production in a cell free system, in a dose-dependent manner. However, CDE had no effect on cytochrome c reduction in human neutrophils stimulated with fMLF or PMA, suggesting that it does not affect NADPH oxidase activity or does not scavenge superoxide anions (O_2_^•-^) as previously described for other medicinal plant extracts such as *Punica granatum* [34] and *Myrtus communis* [35].

The oxygen consumed by neutrophils is enzymatically converted to O_2_^•-^ by univalent transfer of 2 electrons from the cell NADPH [36]. This reaction is catalyzed by NADPH oxidase. The O_2_^•-^ is the source of other ROS such as H_2_O_2_ and the highly toxic hydroxyl radical OH^•^ [36]. ROS may induce several biochemical lesions, including lipid peroxidation, cell membrane disruption, oxidation of sulfhydryl groups, and DNA mutation [37]. More importantly, the present data clearly demonstrated that CDE protects against ROS attacks by scavenging the H_2_O_2_ molecules. Furthermore, other authors reported that this protection might also be provided by inhibition of the myeloperoxidase activity in myrtle berries seeds extract [35].

In vivo, we showed that acute EtOH administration significantly decreased the number of WBC and PLT. Our results partially corroborated those of Kawashima et al. [38] who demonstrated that the administration of 2.0 g/kg of b.w. of ethanol significantly increased the number of neutrophils, basophils, monocytes, and total WBCs without changing the number of PLTs. EtOH had no effect on erythrocytes number or haemoglobin (Hb) and hematocrit (Ht) levels (data not shown). These results are in agreement with previous findings, suggesting that a single administration of ethanol (4 g/kg b.w.) to rats markedly increased the number of natural immunity cells without changing the number of acquired cells [38]. In contrast, chronic administration of alcohol significantly reduced the erythrocytes, WBC, and PLT numbers, Hb concentration, Ht value, mean corpuscular haemoglobin (MCH), and mean corpuscular haemoglobin concentration (MCHC) when compared to control group [39]. However, these discrepancies may be due to the ethanol dosages, the route of administration as well as the period of treatment [40]. The increase in WBC might be due to the marker activation of defence and immune systems and showed that there were inflammations in the tissues [41]. More importantly, we demonstrated in the present work that subacute CDE pre-treatment abrogated all ethanol induced haematological parameters disturbances. The protection offered by CDE against WBC or PLT decrease might be due to its antioxidant [42] and anti-inflammatory [23] properties.

These findings also showed that alcohol administration clearly induced erythrocytes lipoperoxidation increase, sulfhydryl groups decrease, and depletion of antioxidant enzyme activities such as SOD, CAT, and GPx. Ethanol-induced tissue oxidative stress was widely documented in many organ systems such as liver, kidney, heart, brain, and erythrocytes [42–46]. Alcohol consumption can lead to oxidative stress through mechanisms associated to EtOH metabolism that generates ROS [47]. However, ROS production associated to the alcohol-induced depletion of antioxidant enzymes can reduce cellular antioxidant defence capacity, leading to oxidative stress status [48]. More importantly, EtOH-induced erythrocytes oxidative stress has been shown to be attenuated by subacute chamomile pretreatment. EtOH-induced erythrocytes oxidative stress has been previously shown to be attenuated by caffeic acid [49], *Gymnema montanum* [48], beta-carotene [50], olive oil [51], and *Opuntia ficus indica* [46]. The erythroprotective effects of CDE against oxidative stress induced by acute ethanol administration may be due to its richness in biomolecules with significant antioxidant capacity such as phenolic compounds. According to the study done in our laboratory, phytochemical studies of CDE revealed the presence of high concentrations of total polyphenols, total flavonoids, and condensed tannins. The use of HPLC-PDA-MS allowed to the identification of gallic acid, protocatechuic acid, chlorogenic acid, cafeic acid, cafeoylquinic acid, salicylic acid, quercetin, quinic acid derivative, hydroxybenzoic acid-O-hexoside, 5,7,4’-Trihydroxy-6,3’-dimethoxyflavone [52]. These molecules are the primal source of antioxidant ability of this plant that act as scavengers of free radicals [53].

We next sought to determine the putative involvement of some intracellular mediators in EtOH and CDE modes of action. We firstly showed that alcohol administration significantly increased the plasma (data not shown) and erythrocytes H_2_O_2_, free iron, and calcium levels. The implication of these intracellular mediators in the EtOH mode of action has been previously well documented in the hepatic tissue [54–57]. Furthermore, both iron and H_2_O_2_ accumulation catalyzed the highly toxic OH^•^ production via the Fenton reaction leading to membranes lipoperoxidation and enhancement of its permeability to calcium [58]. Interestingly, our data showed that subacute CDE pre-treatment significantly attenuated all EtOH-induced intracellular mediators’ disturbances.

The possible mechanism by which CDE exerts its beneficial effect on erythrocytes could be its ability of chelating free iron and scavenging H_2_O_2_, leading to calcium homeostasis as previously proposed for other extracts rich in phenolic compounds as grape seeds and skin extracts [59, 60], myrtle berries seeds extract [35] and *Myrtus communis* leaves essential oils [61]. CDE could also act on calcium channels known for their implication in iron-overload disorders [62]. Further works are needed to assess the effect of chamomile extract on hepcidin, an iron shuttling protein, known for its implication in the pathogenesis of iron overload [63].

## Conclusions

In the present work, we clearly demonstrated that subacute CDE pre-treatment exerts protective effects against ethanol-induced haematological parameters disturbances and erythrocytes oxidative stress. The beneficial effect of CDE may be explained owing to its ROS scavenging properties and opposite effects on some intracellular mediators such as H_2_O_2_, free iron, and calcium.

## Abbreviations

CAT: Catalase
CDE: Chamomile decoction extract
EtOH: Ethanol
GPx: Glutathione peroxidase
H_2_O_2_: Hydrogen peroxide
MDA: Malondialdehyde
PLT: Platelets
ROS: Reactive oxygen species
SOD: Superoxide dismutase
WBC: White blood cells
